# A novel method for modeling tonic and phasic pupil dynamics in humans

**DOI:** 10.3758/s13428-025-02755-7

**Published:** 2025-07-25

**Authors:** Matthias Mittner, Josephine Maria Groot

**Affiliations:** Institute for Psychology, UiT – The Arctic University of Norway, Hansine Hansens veg 18, Tromsø, 9019 Norway

**Keywords:** Pupillometry, LC/NE, Norepinephrine, Tonic, Phasic, Deconvolution, Algorithm

## Abstract

The human pupil is a widely used physiological metric in psychology and neuroscience. Changes in pupil diameter (PD) are thought to reflect changes in locus coeruleus-norepinephrine (LC/NE) activity, which is associated with cognitive and behavioral optimization. Here, we present a novel algorithm to decompose the pupil signal into its tonic and phasic components. We evaluate the utility and validity of the algorithms using both artificially generated data and an existing dataset from a fast-paced finger-tapping task. Results show that the novel algorithm outperforms traditional approaches on simulated data. We further demonstrate that our algorithm provides more conclusive evidence for relationships between mind wandering reports and pupil predictors compared to traditional window-averaging. Finally, we demonstrate that the novel and traditional estimates contain distinct information regarding neuroimaging correlates and task performance.

The dilation of the human pupil is a physiological metric commonly used by researchers in the fields of psychology and neuroscience. Already in the mid-20th century, it was revealed that pupil responses were altered by cognitive processes such as memory load (e.g., Kahneman & Beatty, 1966). Since then, the pupillary signal has been extensively investigated in the context of various psychological domains, including emotion (Bradley et al., 2008), decision-making (Gee et al., 2017), and language (Zellin et al., 2011). Previous work furthermore demonstrates that changes in pupil diameter (PD) relate to changes in attentional state and mind wandering (Mittner et al., 2014; Unsworth & Robison, 2016). Together, these findings have highlighted pupillometry as a useful tool for gauging covert psychological processes that are otherwise challenging to monitor on short timescales.

The musculature surrounding the iris is controlled by two pathways (Grujic et al., 2024; Mathôt, 2018; Steinhauer et al., 2022). The fast and strong $pupil$ $constriction$ in response to light is mediated by acetylcholine innervating the iris sphincter muscle through the Edinger-Westphal nucleus in the parasympathetic pathway. Instead, $pupil$ $dilation$ in response to psychosensory stimulation is thought to be regulated by the sympathetic nervous system. While complex and not completely understood, this pathway is predominantly characterized by norepinephrinergic (NE) projections from the locus coeruleus (LC) and hypothalamus that ultimately activate the iris dilator muscle. Accordingly, changes in PD observed during stable lightning conditions are taken to primarily reflect changes in LC/NE-mediated activity (Mathôt, 2018).

In recent years, the role of LC/NE-dependent neuromodulation in cognitive and behavioral optimization is increasingly recognized (Aston-Jones & Cohen, 2005; Mittner et al., 2016). A particularly influential theory, the adaptive gain theory (AGT), is rooted in the finding that the LC operates in two distinct firing modes – tonic or regular discharge rates and phasic bursts of activity – that play specific roles with respect to modulating the sensitivity, or $gain$, of cortical computations and consequently optimization of behavior (Aston-Jones et al., 1999; Aston-Jones & Cohen, 2005). Indeed, changes in PD were found to correlate with tonic LC discharge rates recorded intracranially in non-human primates (Joshi et al., 2016; Rajkowski, 1993) and fMRI-BOLD signal from the LC in humans (Murphy et al., 2014), and show the expected Yerkes–Dodson relationship with behavioral performance (Gilzenrat et al., 2010; Jepma & Nieuwenhuis, 2011; Van Den Brink et al., 2016). These findings have opened opportunities for researchers to non-invasively derive tonic and phasic LC/NE activity from baseline and event-related pupil dilation. A theoretical implication of the AGT is that tonic and phasic patterns of LC firing carry independent information regarding the underlying gain state of the neuronal population. Therefore, in order to interpret PD as a proxy for LC/NE dynamics, one needs to analytically separate tonic and phasic components of the pupil signal. In this regard, we argue that contemporary methods exhibit several shortcomings, which we seek to overcome with the presented novel algorithms that are designed to decompose the pupil signal into its constituent tonic and phasic components. In a typical event-related task design, the mean PD over a prestimulus window (e.g., -200 to 0 ms) is often taken as baseline, while pupil responses are calculated as the baseline-corrected mean or peak dilation in a poststimulus window (e.g., 1000—2000 ms). Given that psychosensory pupil responses resolve back to baseline in the order of seconds (~2–3 s, Hoeks & Levelt, 1993; Mathôt, 2018), relatively long inter-stimulus intervals are ideally required to prevent an accumulation of evoked dilatory responses that carry over into the subsequent trials’ prestimulus windows. This creates substantial constraints on the task design and hinders interpretability of underlying tonic changes in faster-paced paradigms, where the artificial build-up of evoked transients is likely conflated with increases in baseline. Traditional approaches are furthermore restrictive in the sense that continuous data are lost. Instead, modeling ongoing tonic fluctuations separately from phasic responses allows comparing dynamic ranges across different experimental conditions (e.g., resting-state and cognitive demand) and may be of theoretical interest for researchers studying the temporal evolution of mental processes or latent states that tend to arise and dissipate more spontaneously.

In summary, we propose a novel modeling approach that assumes that the pupil signal is composed of a slow-fluctuating tonic component and the accumulation of transient dilatory responses that together make up the phasic component. By leveraging the continuous nature of pupillary data, underlying tonic fluctuations are estimated with a Bayesian optimization procedure. To recover the magnitude of psychosensory dilatory responses, we implemented a deconvolution technique based on the canonical pupil response function by Hoeks et al. (1993). Both algorithms are implemented in the open source Python package pypillometry (Mittner, 2020). Here, we evaluate the utility and validity of the algorithms using both artificially generated data and an existing dataset from a fast-paced finger-tapping task (Alexandersen et al., 2022).

## Method

### Model of the pupillometric signal

Assuming the absence of any lighting-related perturbations of the pupil, we assume that the pupillary signal $s(t)$ (where $t$ reflects time) is composed of a tonic component $b(t)$ and a sum of scaled phasic components $\phi _i(t)$ that are elicited by each internal or external event $i\in \{1,\ldots ,N\}$

1 $$\begin{aligned} { s(t) = b(t) + \sum _{i=1}^N \beta _i \phi _i(t-e_i) + \varepsilon (t). }\end{aligned}$$

**Fig. 1:**
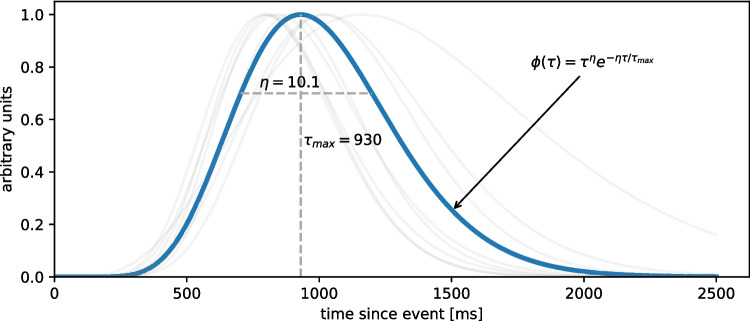
Canonical pupil response function (PRF, Hoeks & Levelt, 1993). *Grey curves* in background illustrate interindividual variability

In this model, $\beta _i$ is a scaling coefficient that determines the strength of the phasic response $\phi _i(t)$, $e_i$ is the onset of each external event and $\varepsilon (t)$ is a zero-mean Gaussian noise term, $\varepsilon (t)\sim \mathcal {N}(0,\sigma ^2)$. We assume the phasic responses to follow the canonical pupillary response function (PRF) as measured by Hoeks et al. (1993) (see Fig. 1) and described as

2 $$\begin{aligned} { \phi (\tau ; \eta , \tau _{max}) = \tau ^\eta e^{-\eta \tau /\tau _{max}} }\end{aligned}$$

which is parametrized by $\eta$ (a parameter modeling the spread of the response function) and $\tau _{max}$ (a parameter modeling the time-to-peak of the response function). Here, $\tau$ is the time relative to the onset of the event at $\tau =0$ and we assume $\phi _i(t)$ to be zero before the onset of the $i$th event.

This model disregards the existence of other potential artefacts in the pupillary signal such as blinks, saccades, or gaze position-related biases. We assume that these artefacts have been removed or interpolated before the application of our algorithm.

### Estimating tonic and phasic pupillary signals

We will now detail a new method to estimate the tonic component $b(t)$ and the phasic components $\beta _i\phi (t)$ from the observed and pre-processed pupillary signal $s(t)$. We recommend that blink-related artefacts are removed and interpolated (e.g., using the method developed by Mathôt et al., 2013), that the signal is low-pass filtered (e.g., using a Butterworth filter with a cutoff frequency of 5 Hz, Alexandersen et al., 2022) and that the signal is corrected for biases related to gaze position (e.g., using the method developed by Hayes & Petrov, 2016). We also suggest, that the signal is scaled to have a mean of zero and a standard deviation of one (Z-scored) as this property is assumed for estimating the tonic component.

#### Estimating the tonic component

Our model in Equation 1 assumes that the signal relaxes to baseline levels in the absence of any external or internal events. Hence, the troughs of the signal are closest to what we assume to be the tonic component. We therefore take the approach of detecting the troughs of the signal and then estimate a smooth curve staying below but close to the troughs. In addition, we prioritize the most “prominent” troughs, i.e., those that are deeper and wider than others, since minor troughs can also be caused by phasic responses. This approach is justified by simulations showing a consistent negative correlation between the prominence of the troughs and the distance between simulated baseline and signal. This correlation (between -0.3 and -0.4) is present across a wide range of simulation settings.

We use the concept of topographic prominence, which is a measure of how much a valley stands out from the surrounding signal. It is defined as the vertical distance between the valley and its highest contour line (Kirmse & Ferranti, 2017) and we assign each of the $\nu \in \{1, \ldots , V\}$ valleys located at timepoints $t_{\nu }$ in our signal its prominence value $p_\nu$. Next, we create a set of $V$ cubic B-spline basis functions $B_\nu (t)$ that are centered around the troughs of the signal ($t_{\nu }$ serve as the control points). We then estimate the tonic component $b(t)$ as a linear combination of these basis functions

3 $$\begin{aligned} { b(t) = \sum _{\nu =1}^V \alpha _\nu B_\nu (t). }\end{aligned}$$

To estimate the coefficients $\alpha _\nu$, we create an objective function that 1) strongly penalizes baseline estimates that exceed the observed signal (i.e., the baseline is forced to stay below the signal $s$) and 2) that prioritizes the most prominent troughs, i.e., it has a high probability to go through high-prominence peaks and a lower probability to go through low-prominence peaks. To define the objective function (Equation 10), we use the asymmetric Laplace distribution to model the likelihood $p_t(s(t)-b(t)|\alpha _\nu )$ of the difference between the estimated baseline $b$ and the pupillary signal $s$ at time $t$:

4 $$\begin{aligned} { p_t(s(t)-b(t)|\alpha _\nu ) = \text {ALD}\left( \sum _\nu s(t) - \alpha _\nu B_\nu (t); \lambda _t, \kappa \right) , }\end{aligned}$$

where the asymmetric Laplace distribution (ALD) is given by

5 $$\begin{aligned} { \text {ALD}(x; \lambda , \kappa ) = \left( \frac{\lambda }{\kappa +1/\kappa }\right) \exp \left( -x\lambda \text {sgn}(x)\kappa ^{\text {sgn}(x)}\right) . }\end{aligned}$$

**Fig. 2:**
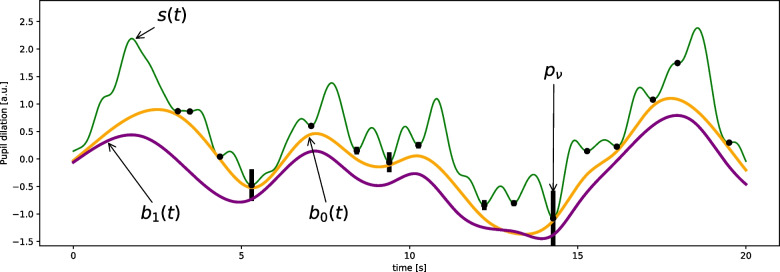
Illustration of the baseline estimation. The troughs (*black dots*) of the pupillometric signal (*green line*) are found and their topographic prominence $p_\nu$ calculated. The baseline that is estimated in two steps ($b_0$ and $b_1$, see Algorithm 3) is encouraged by the objective function to pass close to the high-prominence troughs (*black bars*)

Here, $\lambda$ is the scale parameter, $\kappa$ is the asymmetry parameter and $\text {sgn}(x)$ is the sign function that is $-1$ for $x<0$ and $+1$ for $x\ge 0$. This probability distribution decays exponentially around zero (here, zero corresponds to the pupillary signal $s$) and the rate with which it decays is asymmetric for $x>0$ and $x\le 0$ where the asymmetry is dictated by parameter $\kappa$. This property of the distribution allows to control the probability of the estimated baseline exceeding the pupillary signal, i.e., $\text {Pr}[b(t)>s(t)]$, while simultaneously allowing the distance of the baseline below the signal to vary by topographic prominence. While theoretically, the baseline should never exceed the pupillary signal, for computational reasons, we use a likelihood function that allows the baseline to occasionally exceed the signal. Namely, we specify that 5% of the density of the asymmetric Laplace distribution should be below 0 ($p_a=0.05$). Using the properties of the asymmetric Laplace distribution, we find the asymmetry parameter $\kappa$ to be

6 $$\begin{aligned} { \kappa = \frac{\sqrt{p_a}}{\sqrt{1-p_a}}. }\end{aligned}$$

The parameter $\lambda _t$ is time-dependent and chosen separately for the troughs $\nu$ of the signal as $\lambda _\nu$ and the other parts of the signal, $\lambda _{min}$. For the troughs, the $\lambda _\nu$ are chosen such that the distribution will be narrow when the prominence $p_\nu$ of the trough is high and wide when the prominence is low

7 $$\begin{aligned} { \lambda _\nu = \lambda _{min} + (\lambda _{max}-\lambda _{min})\frac{p_\nu - \min _{\nu '}\left( p_{\nu '}\right) }{\max _{\nu '}\left( p_{\nu '}\right) - \min _{\nu '}\left( p_{\nu '}\right) }.}\end{aligned}$$

Here, $\lambda _{min}$ and $\lambda _{max}$ are tuning parameters that we globally set based on the expected properties of the signal and $\min _i x_i$ and $\max _i x_i$ are operators that calculate the minimum and maximum of the vector $x_i$. Assuming a Z-scaled signal, setting $\lambda _{min}=1$ imposes minimal constraints on the signal where no troughs are present (i.e., the baseline is allowed to fluctuate freely below the signal) and $\lambda _{max}=100$ imposes a strong constraint on the signal where the troughs are very prominent (i.e., the estimated baseline is forced to go near the high-prominence troughs). Formally,

8 $$\begin{aligned} { \lambda _t = {\left\{ \begin{array}{ll} \lambda _\nu & \text {if } t \in \{\nu _1, \ldots , \nu _V\}\quad \text { (Eq. 7)}\\ \lambda _{min} & \text {otherwise}. \end{array}\right. }. }\end{aligned}$$

Together, this defines an effective “corridor” in which the baseline is allowed to fluctuate that is constrained more strongly by the most prominent troughs (see Fig. 2).

Putting together Equation 4 and Equation 7, we can define the likelihood of the estimated difference between baseline and signal given the set of parameters $\alpha _\nu$ as

9 $$\begin{aligned} { p(s-b|\alpha _\nu ) = \prod _{t=1}^T p_t\left( s(t)-b(t)|\alpha _\nu ; \lambda _t, \kappa \right) , }\end{aligned}$$

where $T$ is the number of time points in the signal. We apply Bayes’ rule to obtain the posterior probability of the parameters defining the tonic component given the observed signal as

10 $$\begin{aligned} { p(\alpha _\nu |s) \propto p(\alpha _\nu )\prod _{t=1}^T \text {ALD}\left( s(t) - \sum _{\nu =1}^V \alpha _\nu B_\nu (t); \lambda _t, \kappa \right) , }\end{aligned}$$

where $\kappa$ is determined according to Equation 6 and $\lambda _t$ according to Equation 8. Finally, we place weakly informative priors on the parameters, $\alpha _\nu \sim \mathcal {N}(0, 5)$.

We estimate the optimal coefficients $\hat{\alpha }_\nu$ by maximizing this posterior probability using a variational inference algorithm (Kucukelbir et al., 2017) implemented in the Stan software (Carpenter et al., 2017). The final estimate of the tonic component is then given by the linear combination of the B-spline basis functions with the estimated coefficients $\alpha _\nu$ as in Equation 3. For computational efficiency, the signal can be downsampled before applying the algorithm and then upsampled again after the estimation of the tonic component as long as the sampling rate is sufficient to sample the expected frequencies in the baseline signal. We recommend a sampling rate of at least 10 Hz for the estimation of the tonic component. See Algorithm 1 for a summary of the algorithm.

**Algorithm 1:**
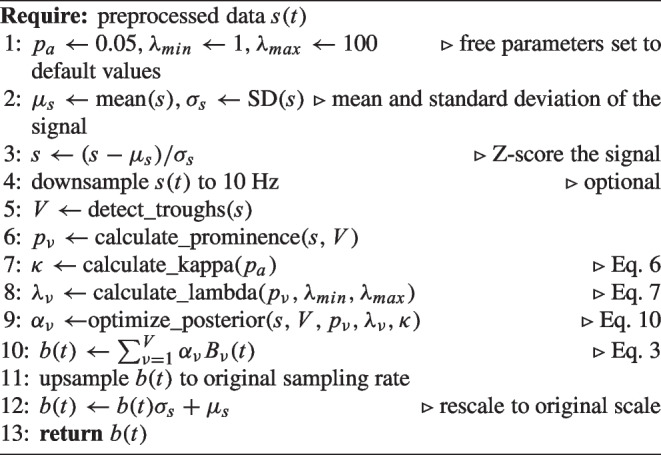
Estimating the tonic component

#### Estimating the phasic component

Given the known onsets of $E$ external events $e_i$ ($i=\{1,\ldots ,E\}$), we estimate the phasic component for these events by subtracting the estimated tonic component from the observed signal and then estimating the phasic component relative to the new baseline

$$\begin{aligned} r(t) = s(t) - b(t). \end{aligned}$$

At each event onset $e_i$, we estimate the phasic component $\beta _i\phi (t; \eta , \tau _{max})$ by fitting the canonical PRF (Equation 2) to the signal in a window around the event onset. For a given set of parameters $\eta$ and $\tau _{max}$, we estimate the scaling coefficients $\beta _i$ using non-negative least squares (NNLS, Bro & De Jong, 1997) which optimizes

11 $$\begin{aligned} { \int _T\left[ r(t) - \sum _{i=1}^E \beta _i \phi (t-e_i; \eta , \tau _{max})\right] ^2dt }\end{aligned}$$

subject to the constraint that $\beta _i\ge 0$ (phasic components are assumed to be positive relative to the baseline). Furthermore, we optimize the parameters $\eta$ and $\tau _{max}$ (these are assumed to be constant for all trials of each participant) using a non-linear optimization algorithm such as the Nelder–Mead algorithm (Nelder & Mead, 1965). The coefficients $\beta _i$ constitute the estimated phasic response for trial $i$. See Algorithm 2 for a summary of the algorithm.

**Algorithm 2:**
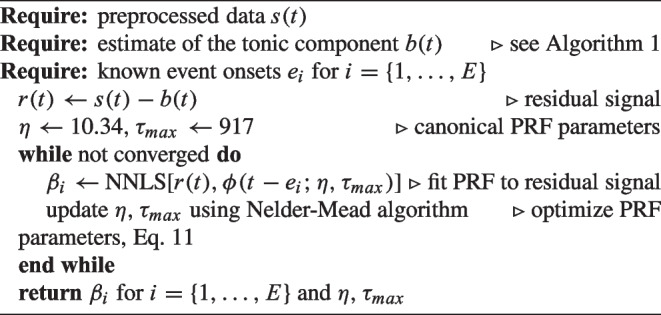
Estimating the phasic component

#### Full algorithm

The full algorithm to estimate the tonic and phasic components of the pupillary signal applies an iterative application of the baseline estimation and the phasic component estimation. We start by estimating the tonic component using the method described above. Next, we subtract the estimated tonic component from the observed signal to obtain the residual signal and estimate the phasic components from the residual signal as described above. We then subtract the estimated phasic component from the original signal and iterate the estimation of the baseline. This baseline is then used in a final step to estimate the phasic responses relative to that new baseline. The algorithm is summarized in the pseudo-code in Algorithm 3.

**Algorithm 3:**
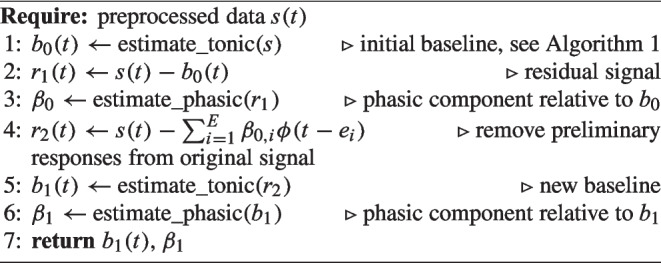
Full algorithm to estimate tonic and phasic components

**Fig. 3:**
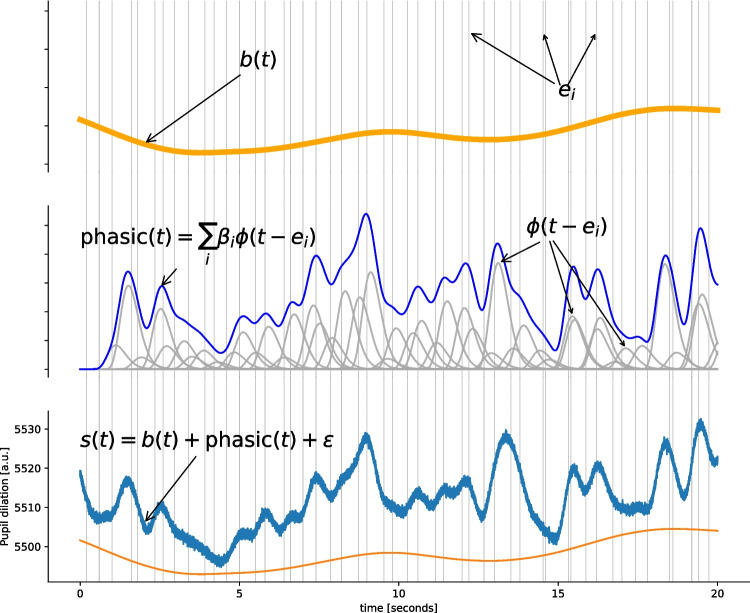
Twenty seconds of simulated pupillometric signal. The tonic model (*top panel*) is combined with the sum of the phasic responses (*middle panel*) located at event onsets (*grey vertical stripes*) to yield the final simulated dataset (*lower panel*)

### Simulated data

To test the performance of our algorithm, we generate artificial data according to our model of the pupillometric signal (Equation 1) for a basic psychological experiment where participants have to respond to presented stimuli over $N$ trials. We generate a slowly fluctuating baseline signal $b(t)$ by filtering white noise with a low-pass filter with a cutoff frequency of 1 Hz. Next, we simulate event-onsets corresponding to external stimuli by creating regularly spaced event onsets separated by specific inter-stimulus-intervals (ISI). Next, we simulate reaction times by drawing random values from a truncated normal distribution with mean $\mu _{RT}$ ms and an SD of $\sigma _{RT}$ ms. The timing of these events is denoted by $e_i$ and both the presentation of stimuli and response is assumed to elicit a phasic response of strength $\beta _i$ and shape determined by parameters $\eta$ and $\tau _{max}$ according to Equation 2. Hence, we model $E=2\times N$ known responses (one stimulus and one response per trial). The strength of the phasic response is set to random values drawn from a normal distribution with mean $\mu _{\beta }$ and SD $\sigma _{\beta }$. The simulated baseline and response functions are summed up and perturbed by zero-mean noise $\varepsilon \sim \mathcal {N}(0,\sigma _\varepsilon )$, see Fig. 3.

Finally, to make the signal more realistic, we simulate a number $E^{*}$ of “internal” (unobserved) events $e^{*}_j$ which are drawn from a uniform distribution $e^{*}\sim \mathcal {U}(0,T)$. For each of these unobserved events, we add an elicited response to the simulated signal as described above. However, the timing of these events will not be available during the estimation and will therefore introduce additional uncertainty over and above the iid noise modeled by $\varepsilon$.

The main quantity of interest for researchers are the trial-by-trial estimates of the tonic and phasic components. We will therefore report the correlation between the true and the estimated values of the tonic and phasic components. As a comparison, we will also report corresponding results for the traditional approach where the baseline is estimated as the average signal in a pre-specified time window before the event onset and the phasic response is estimated as the difference between the average observed signal in a (usually flexibly chosen) time window after the onset of the event and the estimated baseline. We will choose the optimal time window for the traditional approach by selecting a 200 ms window around the peak of the known pupil response, i.e., $[\tau _{max}-100, \tau _{max}+100]$ ms. In actual studies, this time window has to be chosen *a priori* or by inspecting the grand average of the observed signal and selecting a time window that captures the peak of the response. This introduces an additional bias in the estimation of the phasic response and our simulations can therefore be considered to be optimistic for the traditional approach. The time window for the estimation of baseline activity is chosen as $[-200, 0]$ ms before the event onset.

**Fig. 4:**
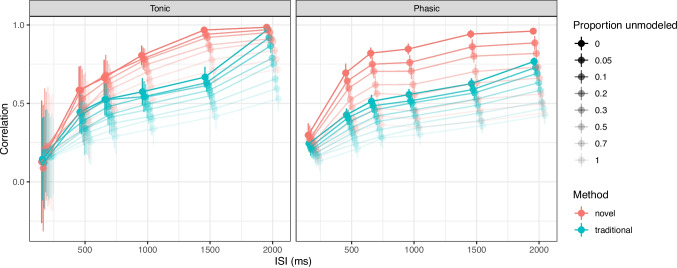
Performance of the algorithm on simulated data depending on the ISI (*x-axis*) and the proportion of unmodeled responses (*shade*). The y-axis displays the correlation between estimated and ground-truth tonic component (*left panel*) and phasic component (*right panel*). Our algorithm (*red*) outperforms the traditional approach (*blue*) in all cases. Both methods become more reliable in the case of longer ISIs and a lower number of unmodeled responses. Error bars are standard deviations across the $n_{rep}$ datasets

**Fig. 5:**
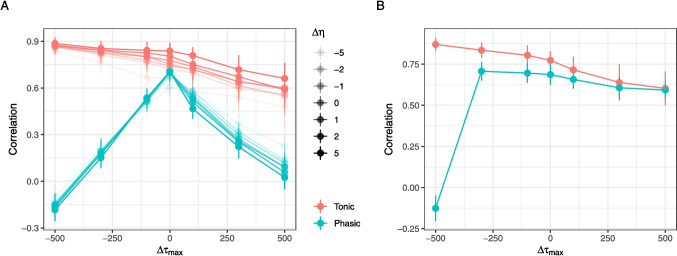
Robustness of the algorithm against misspecification of the PRF parameters $\tau _{max}$ and $\eta$. The *y*-axis displays the correlation between estimated and ground-truth tonic and phasic components. **A** The tonic estimate (*red*) is robust against misspecification of both $\tau _{max}$ (*x*-axis) and $\eta$ (shade) while the phasic component (*blue*) is more sensitive to misspecification of $\tau _{max}$

## Results

### Performance on simulated data

First, we started by evaluating the algorithm’s performance in different hypothetical scenarios where we systematically manipulated the pace of the experiment (the ISI) as well as the number of unknown (internal) responses $E^*$. In this simulation, we simulated $n_{rep}=100$ datasets for each combination of parameter settings (ISI and $E^*$). Each dataset contained $N=100$ trials and was simulated with $\mu _{RT}=300, \sigma _{RT}=100$ and $\eta =10.35, \tau _{max}=917$. The results of this simulation are presented in Fig. 4. Unsurprisingly, both algorithms perform better with longer ISIs and fewer unmodeled responses. The reason for this is that the longer ISIs allow the tonic component to relax to baseline levels and the phasic responses to resolve back to baseline. The traditional approach is more sensitive to the presence of unmodeled responses as these will inflate the estimated baseline and thereby reduce the estimated phasic response. Even for fast-paced with an ISI of 700 ms, the novel algorithms performs quite well where the correlation with the ground truth is around 0.7 depending on the number of unmodeled events. The number of unmodeled events affects the estimation of the phasic response more than the tonic component.

Next, we wished to investigate the effect of misspecifying the parameters of the pupil response function $\eta$ and $\tau _{max}$ on the performance of the algorithm. We simulated datasets using the same parameters as in the previous simulation setting the ISI to 1000 ms and the proportion of unmodeled events to 10%. We then simulated datasets with different values of $\eta$ and $\tau _{max}$ and estimated the tonic and phasic components using the novel algorithm but without estimating these parameters freely (i.e., we fixed them to the canonical values of $\eta =10.35$ and $\tau _{max}=917$). Therefore, each of the datasets was misspecified by $\Delta \eta$ and $\Delta \tau _{max}$. The results of this simulation are presented in Fig. 5 A. The estimation of the tonic component is quite robust against misspecification of both $\eta$ and $\tau _{max}$, while the estimation of the phasic component is more sensitive to misspecification of $\tau _{max}$ but not $\eta$: With increasing absolute distance of the simulated $\tau _{max}$ from the canonical value, the correlation between the true and estimated phasic response decreases. This is expected as the shape of the phasic response is determined by $\tau _{max}$ and the algorithm will therefore have a harder time estimating the phasic response when the true $\tau _{max}$ is far from the canonical value.

We therefore investigated how well the algorithm can compensate for that negative effect of misspecification by estimating $\eta$ and $\tau _{max}$ freely. This is crucial, since the shape of the PRF varies quite widely between individuals (Hoeks et al., 1993). We repeated the previous simulation but now estimated $\eta$ and $\tau _{max}$ freely during each fit. The results of this simulation are presented in Fig. 5 B. The estimation of the phasic component is now robust against misspecification of $\eta$ and $\tau _{max}$ and only in extreme misspecifications ($\Delta \tau _{max}=-500$ ms) does the performance of the algorithm suffer dramatically. This demonstrates the importance of estimating the shape of the PRF freely in order to obtain reliable estimates of the phasic response.

### Application to real data

We also wished to demonstrate the utility of the algorithm on real data. We therefore applied the algorithm to a dataset from a fast-paced finger-tapping task (Boayue et al., 2021) and analyzed how the estimated relationship between tonic and phasic pupillary variables and behavior would be affected by using our novel algorithm compared to the traditional approach.

#### Experimental task and data

Data were previously analyzed and published as part of our recent study investigating the effects of transcranial direct current stimulation (tDCS) on mind wandering with experience sampling, pupillometry, and EEG (Alexandersen et al., 2022). Binocular pupillometric data were collected from 100 healthy volunteers with a desktop-mounted EyeLink 1000 tracker (SR Research) at 500-Hz sampling rate. During the task, participants fixated on a centered white cross on a grey background while tapping left and right index fingers in rhythmic synchrony with an ongoing auditory cue presented every 750 ms. While keeping in rhythm with the cue, participants were tasked with maximizing the entropy of their tapping-sequences by randomizing the order of left and right presses – thereby recruiting executive and attentional resources to avoid repetitions in response patterns. Materials, data, and code are online available at https://osf.io/cv24f/.

#### Extracting tonic and phasic features

Data were selected from the eye with the least missing samples. All preprocessing steps as well as estimation of tonic and phasic components were performed in Python using the pypillometry (Mittner, 2020) package. Blinks were detected with manually fine-tuned, subject-specific velocity profiles and linearly interpolated. Data were temporally filtered with a 5 Hz lowpass Butterworth filter. The tonic and phasic components of the preprocessed pupil signal were estimated with the algorithms presented above. We extracted single-trial tonic features as the tonic value at stimulus onset (i.e., each auditory cue). The close temporal proximity of stimuli and responses resulted in multicollinearity among estimated coefficients, we therefore calculated single-trial phasic features as the sum of all coefficients in the (-200, 200) ms window around each stimulus. For comparison, we extracted features in a more traditional approach by taking the mean preprocessed pupil signal in the (-200, 0) ms prestimulus window as the baseline and the difference between the mean signal in the (500, 700) ms poststimulus window and the baseline as the evoked pupil response.

**Fig. 6:**
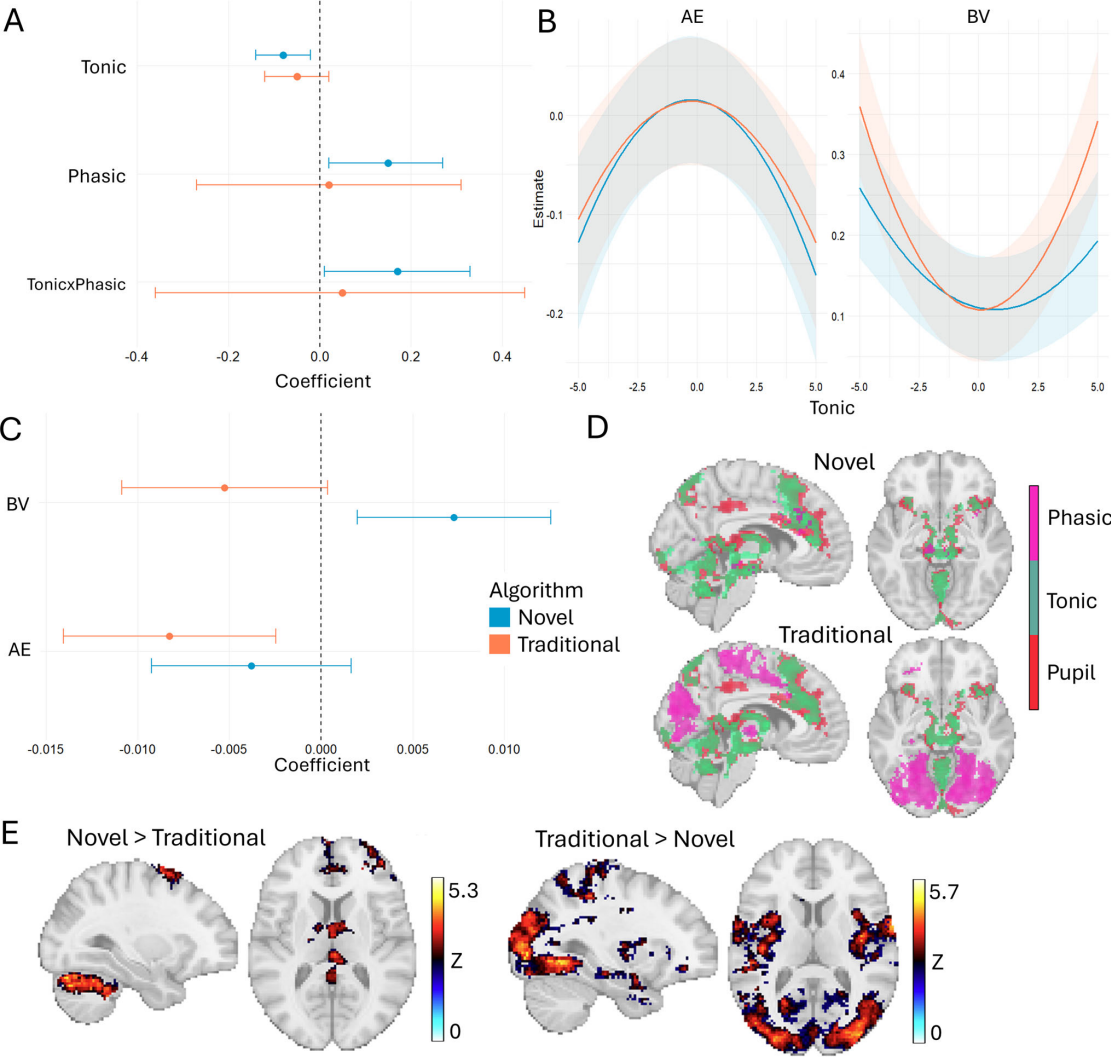
Results for comparing our novel algorithm to traditional window-averaging on real data. **A** Regression coefficients for two models regressing self-reported mind wandering on the pupillary features. **B** Results for the quadratic tonic predictor and **C** the linear phasic predictor from four mixed-effect models with single-trial behavioral variability (“BV”) and approximate entropy (“AE”). **D** Results from whole-brain GLMs on fMRI data from 21 subjects (Groot et al., 2022), overlaying the thresholded *z*-statistical spatial maps for the preprocessed pupil signal and tonic and phasic single-trial regressors resampled to the resolution of the fMRI timeseries. **E** Results from a FLAME analysis directly comparing the first-level GLM contrasts for novel and traditional estimates of phasic responses

All single-trial features – i.e., both those modeled with the novel algorithms and traditional window-averages – were then standardized (z-scored) to account for individual differences in pupil size. The correlation between modeled tonic and phasic features was low (mean $r$ = .07, $SD$ = .09), while traditional baseline and evoked features were moderately correlated (mean $r$ = -.26, $SD$ = .05). These relationships may be illustrative for the way in which the novel algorithm enforces the tonic estimate as a lower envelope of the pupillometric signal that is largely independent from accumulating phasic transients, while traditional baseline estimates are more likely inflated, thereby negatively affecting the estimated magnitude of the evoked response relative to the baseline.

#### Relationships to task-related and task-unrelated processes

We tested both sets of features (novel vs. traditional) for relationships with self-reported mind wandering as measured on a 4-point Likert scale, expecting that features extracted with the novel algorithm would result in stronger effects compared to traditional window-averaging. We averaged single-trial features across the 25 trials (18.75 s) preceding a total of 2839 mind wandering probes and used Bayesian hierarchical ordered-probit regression models with subject-level random intercepts; one for novel and one for traditional predictors. We report the posterior means and highest-density intervals (HDI) for models with mind wandering report as dependent variable and single-trial tonic and phasic features and their interaction as independent variables (note that this differs from Alexandersen et al., 2022 which also included block effects in the model). Posterior means and 95% highest-density intervals (HDI) are shown in Fig. 6A, demonstrating more conclusive evidence for relationships between mind wandering reports and tonic and phasic pupil predictors extracted with the novel method compared to traditional window-averaging.

Additionally, we tested relationships between pupil features and behavioral performance in terms of behavioral variability (BV) and approximate entropy (AE), where poorer task performance is characterized by higher BV (i.e., less rhythmicity in tapping onsets) and lower AE (i.e., less entropy in the tapping sequence). Theoretically, tonic dynamics should follow a Yerkes–Dodson relationship with performance, where both low and high values suppress phasic bursts of activity and interfere with optimal performance. We used a linear mixed-effects model to investigate the relationships between performance indicators as dependent variables (fitting separate models for BV and AE) and pupil predictors as independent variables (fitting separate models for novel and traditional estimates), with a quadratic term for tonic features to model non-linear relationships. Single-trial BV and AE were calculated across the previous 25 trials. We observed the expected Yerkes–Dodson relationships for both novel and traditional tonic estimates and both performance indicators, indicating that poorer task performance was associated with both small and large tonic pupil size (Fig. 6B).

However, linear relationships between task performance and the magnitude of phasic responses were dissociable for novel and traditional estimates, as indicated by effects with opposite directions for BV and a negative association with AE only for the traditional method (Fig. 6C). We interpret this finding as a consequence of our algorithms’ successful uncoupling of the separate contributions that auditory stimuli and behavioral responses have on the pupillary response. During periods of low BV, these responses overlap more strongly and create a spurious negative relationship with BV when using the traditional approach, which lacks the ability to isolate them. Only by separately estimating the phasic responses to both stimulus and behavior can the genuine positive relationship between BV and phasic response be revealed.

#### Neural correlates

In our recent fMRI study, we collected and analyzed data from a similar finger-tapping task to investigate neural correlates of pupillary features, mind wandering, and behavior (Groot et al., 2022). Here, we reanalyzed these data to compare the neural correlates of novel and traditional pupil features using FSL FEAT to fit three generalized linear models (GLM) to the preprocessed fMRI timeseries. All GLMs included boxcar regressors for stimuli and response onsets, nuisance regressors (e.g., motion parameters), and either (i) the preprocessed pupil signal, (ii) tonic and phasic features estimated with the novel algorithm, or (iii) tonic and phasic features estimated from traditional window-averaging. To create the pupil regressors, single-trial features (novel and traditional) were resampled to the resolution of the fMRI timeseries with nearest-neighbor interpolation. Second-level analyses were performed with FLAME and groups of voxels with $Z$ > 2.3 were cluster-thresholded at $p<.05$.

We first compared the results from the GLMs for novel and traditional features to the statistical map of the preprocessed pupil signal (Fig. 6D), showing highly spatially overlapping patterns for novel and traditional tonic estimates but strongly dissociable spatial patterns for novel and traditional phasic estimates. That is, both the novel tonic component and traditional prestimulus estimate correlated with fMRI activity in brainstem areas and anterior cingulate cortex (ACC). Instead, traditional phasic features correlated more widespread to visual and medial frontal cortices compared to novel phasic estimates. We directly compared both statistical maps in a contrast analysis, demonstrating that compared to traditional estimates, novel phasic responses were more focalized to cerebellar and superior frontal regions (Fig. 6E). Given that the task paradigm has no visual stimuli and task performance relies heavily on motor planning and execution, neural correlates in these regions could be interpreted as better reflecting task-specific processes.

## Discussion

Pupillometry has become an increasingly popular tool in psychology and neuroscience and is widely used to measure covert mental processes such as attention, decision-making, and emotion in humans and primates. Here, we have presented new algorithms for analyzing pupillometric data that decompose the continuous pupil signal into tonic and phasic components that can be of separate theoretical interest.

We observed that, compared to the more traditional use of signal averages in prestimulus and poststimulus windows, modeled estimates of tonic and phasic pupil components based on our novel algorithm provided overall better recovery using simulated data. The novel method was less sensitive to increased numbers of spontaneous, “task-unrelated” dilatory events and retained strong to moderate correlations with the simulated ground truth, even at sub-second inter-stimulus intervals. Furthermore, the novel method outperformed the traditional method on experimental data from a fast-paced finger-tapping task, when assessing evidence for relationships with self-reported mind wandering. [In particular, the method produced more precise estimates (as evidenced by narrower HDIs) and was able to uncover a positive effect between BV and phasic pupillary response that was occluded by the traditional methods’ failure to account for the separate effect of stimulus and behavioral response], suggesting that the novel method was more successful at capturing drifts in attentional focus. Reanalysis of simultaneous fMRI-pupil data from a similar finger-tapping task indicated that novel and traditional tonic estimates displayed spatially overlapping neural correlates, whereas spatial maps for novel versus traditional phasic estimates showed a pronounced dissimilarity. Together, these findings may indicate that the magnitude of phasic responses based on our modeling algorithm contains information regarding the underlying behavioral state and fMRI activity that can be dissociated from the signal average in a fixed poststimulus time window.

The vast majority of research with pupillometry in humans has relied on averaged signals in predefined (prestimulus and poststimulus) windows. Our algorithms add to a relatively small repertoire of alternative model-based deconvolution techniques (e.g., Wierda et al. (2012); Gee et al. (2014); Knapen et al. (2016)) and provides a completely novel method for detailed modeling of continuous pupillary data. This approach has several advantages over more standard approaches, among which eliminating the need for arbitrary preselected peak windows and single-trial baseline correction, as well as the ability to fit individually optimized pupil response curves. These advantages may be especially beneficial when administering relatively fast-paced experimental paradigms, where there is a risk of accumulating phasic responses that may affect baseline estimations. However, there are several directions for future development that could further improve the estimation of these components. One future goal is to enable the algorithm to exclude missing or interpolated data rather than relying on processing the reconstructed signal by interpolation methods. Signal reconstruction can potentially introduce artifacts that can systematically affect the estimation of the phasic component. For example, it is possible that an experimental manipulation will affect both blinking frequency and dilatory pupil responses. In such cases, linearly interpolating blinks carries the danger of introducing a systematic bias between conditions in the estimation of the phasic component for the condition that has more blinks, as the interpolated signal will suppress any potential phasic responses. Given that our approach models the pupillary signal with a probabilistic model at each sample point, it is possible to exclude segments of missing data from the estimation or even include a separate predictive model of missing data in the approach.

Another important future direction concerns the extension of our approach to include an experimental design specification. Currently, our algorithm estimates the magnitude of individual events completely independently for each trial. Since researchers are often interested in comparing the pupillary response to different experimental conditions or types of stimuli, the contrasts have to be made *post-hoc* based on statistically comparing the estimated responses between conditions (e.g., with a $t$ test). A better approach would be to include the type of event as a parameter in the model and estimate different PRFs for different types of events. For example, both the magnitude and the parameters determining the shape of the PRF $(\eta ,\tau _{max})$ could be separately estimated and the strength of single-trial responses could be constrained by group-level distributions as done in hierarchical mixed-effects models. This would allow for a more direct comparison of the estimated responses between conditions and would also allow for a more fine-grained analysis of the pupillary response to different types of stimuli.

Finally, the algorithms currently refrain from making assumptions regarding the temporal evolution of phasic responses as well as the relationship between tonic levels and phasic responses. It is typically assumed that the relationship between tonic and phasic components follows a Yerkes–Dodson (inverse U-shaped) curve. This could be modeled by including a temporal constraint on the PRF that would allow for the phasic response to be stronger when the tonic level is further away from an optimal level. It is also feasible to include other, more generic temporal constraints on the estimation of the pupillary responses, for example, correlation among neighboring events, or that an event’s response depends on a preceding event. Such constraints could be implemented by including autoregressive terms in the model or by using a hierarchical model that constrains the estimated responses to be similar across trials.

We have shown that our algorithm provides advantages over traditional approaches when estimating tonic and phasic components of the pupillary signal. However, it is important to note that the applicability and performance of the novel method relative to more traditional approaches will depend on the objectives and limitations of the research question and design. Here, we argue that disentangling tonic and phasic components of pupil dynamics is more principally aligned with the proposed functional modes of the noradrenergic modulatory system, that are posited to play distinct roles in the optimization of behavior (Aston-Jones & Cohen, 2005). As such, this approach may provide new opportunities toward applying and validating influential theories such as the AGT in humans. This may be especially fruitful when interested in global attentional phenomena including mind wandering (e.g., Mittner et al., 2016).

Based on the theoretical assumptions outlined previously, we assume that arousal-related phasic pupil activity is primarily LC-mediated and is therefore treated by the algorithm as dilatory in nature. It is important to note that we designed and tested the algorithms on an equiluminant cognitive task involving auditory stimuli. We expect that, for visual paradigms, event-locked pupil constructions in response to light (e.g., the pupil light reflex) should lead to a constant offset or scaling of the phasic response that will not impact the algorithms performance or outcomes, if stimuli are equiluminant. However, variations in the magnitude of pupil constrictions due to uncontrolled changes in luminance or other physical stimuli attributes, are not accounted for by the algorithms and may effect the resulting model estimations. We therefore recommend that users carefully control for such potential instances (e.g., luminance changes) in their experimental designs. Critically, the algorithms are not designed to handle cases where the change in pupil size from baseline is negative, as this violates the main assumption of the model. It is therefore imperative that users first visually inspect their data, to ensure that the pupil response of interest is primarily a dilatory response, before applying the algorithms in order to avoid misleading results.

Crucially, a growing body of evidence warrants caution when studying the relationship between the pupil and LC/NE-dependent neuromodulatory dynamics. While there exists a correlation between LC spiking activity and pupil diameter, there is substantial variability in this relationship that moreover seems strongly modulated by global brain state, such as decision biases (Megemont et al., 2022). In addition, besides norepinephrine, diverse brain regions and neuromodulators are likely involved in controlling pupil size, including the cholinergic and serotonergic systems (Cazettes et al., 2021; Joshi et al., 2016; Reimer et al., 2016). Given that the neurobiological pathways that link these systems are currently incompletely understood, we recommend to exercise prudence when interpreting pupil as an index for LC/NE-related activity.

In summary, we have described novel algorithms for the decomposition of continuous pupillary data into a slow-fluctuating tonic component and transient dilatory responses to psychosensory stimulation that constitute a phasic component, and have validated its performance on both simulated and real data from a cognitive experiment with auditory stimuli. The algorithms are implemented in the open source Python package pypillometry (Mittner, 2020) available at https://github.com/ihrke/pypillometry. The package provides easy-to-implement functions for preprocessing (e.g., blink identification and removal, temporal filtering, and normalization), visualization, and modeling of pupil dynamics that can be applied to a wide range of research settings and designs that include pupillary data.

## Data Availability

All data used in this study are publicly available and included as part of the pypillometry package. The package is freely available on GitHub under the MIT license at https://ihrke.github.io/pypillometry/.

## Code availability

The code used in this paper is published as part of the Python package pypillometry which is freely available on GitHub under the MIT license at https://ihrke.github.io/pypillometry/.

## Declarations

### Open Practices Statement

Materials and analysis code are available at https://ihrke.github.io/pypillometry/.
